# Risk of venous thrombosis and pulmonary embolism in older in-patients with mental illness: systematic review

**DOI:** 10.1192/bjb.2025.9

**Published:** 2026-02

**Authors:** Damodar Chari, Tamara Chithiramohan, Ina Sawhney, Elizabeta B. Mukaetova-Ladinska, Lucy Beishon, Hari Subramaniam

**Affiliations:** 1 Mental Health Services for Older People, Leicestershire Partnership NHS Trust, Leicester, UK; 2 School of Psychology and Visual Sciences, University of Leicester, UK; 3 Department of Geriatric Medicine, University Hospitals of Leicester NHS Trust, Leicester, UK; 4 Department of Cardiovascular Sciences, University of Leicester, UK

**Keywords:** Antipsychotics, in-patient treatment, mortality and morbidity, old-age psychiatry, risk assessment

## Abstract

**Aims and method:**

Venous thromboembolism (VTE) is a fatal condition affecting older people. This study aims to identify specific risk factors for VTE in older psychiatric in-patients within mental hospital settings. Using predefined search terms, we searched five databases to capture studies evaluating risk factors associated with the occurrence of deep vein thrombosis and pulmonary embolism in older psychiatric in-patients.

**Results:**

Thirteen studies were identified, and a narrative synthesis performed. Increasing age was a consistent risk factor for VTE. Diagnosis and psychotropic medication use were inconsistent. Depression, catatonia and use of restraint in people with dementia were associated with higher risks.

**Clinical implications:**

Older psychiatric in-patients differ from medical and surgical in-patients in their risk profiles. Screening tools used in general hospital patients are of limited use among older adults in psychiatric hospital settings. An exclusive screening tool to identify VTE risk factors in older psychiatric in-patients is needed.

Venous thromboembolism (VTE) is potentially a fatal condition resulting in one in five deaths in people who are not treated for the condition It is one of the leading preventable causes of death globally, with a 1-year mortality rate of about 22%.^1,2^ It is estimated to affect nearly 10 million people worldwide.^3^ VTE-related morbidity is associated with a poorer quality of life and greater healthcare costs.^4^ VTE has an incidence of between 2 and 12% among older people within psychiatric in-patient settings.^5,6^ VTE includes deep vein thrombosis (DVT) when a thrombus is formed within the veins of lower limbs, and pulmonary embolism, a serious consequence of DVT. Further, 43% of medically ill patients develop a silent pulmonary embolism and 7–33% have a fatal pulmonary embolism.^7–9^ Although precise estimates of death among older psychiatric in-patients within mental health settings is not known, this is likely to be significantly higher than the above-mentioned fatality rates of one in five among all VTE-related deaths.

## Risk factors

Surgical risk factors for DVT are well known, and the knowledge of medical risk factors is growing.^10^ However, much less is known about risk factors for the development of DVT in psychiatric patients, particularly in older psychiatric in-patients. There is some evidence to link VTE with both psychotropic drugs and mental illness. Antipsychotics are reported to increase risk of VTE three- to four-fold.^11,12^ Patients who have been physically restrained or have a diagnosis of dementia are also more likely to develop VTE.^13–15^ Additionally, many psychiatric in-patients have comorbidities that increase their risk of VTE.^16^ Although there may be an acute decline of mobility in surgical patients, there is also a progressive reduction of mobility in psychiatric patients with severe mental illness.^15^ This reduced mobility may predate their hospital admission and their functioning within psychiatric hospital settings.^16^ International guidelines recommend that all mental health in-patients should have a VTE risk assessment on admission.^17^

We undertook a systematic review of the existing literature to establish current understandings of the specific risk factors and outcomes for VTE in older psychiatric in-patients, with the aims of (a) identifying risk factors for VTE in older psychiatric in-patients, (b) determining whether they are distinct from patients in general medical settings and (c) identifying any risk assessment tools that are specific for this patient group.

## Method

### Searches

We searched the following databases: Medline (1946–2023), EMBASE (1947–2023), PsycINFO (1984–2023), CINAHL (1976–2023) and the Cochrane Library (1993–2023) using predefined search strategy (Supplementary Appendix 1 available at https://doi.org/10.1192/bjb.2025.9). The search strategy was developed in conjunction with a librarian (Stuart Glover, Librarian at University Hospitals of Leicester NHS Trust). Searches were restricted to studies in English or where the translation in English was available. In addition, reference lists and citation indices of relevant papers were searched, and the PubMed related articles feature used to identify any further articles. This review was registered with PROSPERO under the registration number CRD42022352798.

Abstracts of articles identified by the search were screened independently by two reviewers (T.C., D.C.) and then full texts were independently reviewed (T.C., D.C.). There was only one disagreement within the studies obtained through data searches in relation to a study by Schmidt et al,^18^ and this was resolved after a discussion with a third reviewer (I.S.).

### Study selection

We included evidence from multiple study types, including longitudinal studies, randomised controlled trials, non-controlled trials, cohort, case–control, cross-sectional studies and observational studies. We included studies on older patients who developed DVT and pulmonary embolism in psychiatric in-patient settings. We defined ‘older’ as a person aged ≥65 years. We recorded the factors significantly associated with the occurrence of venous thrombosis in these studies.

### Participants/population

We included studies of older people who had a documented DVT or pulmonary embolism in their records or had DVT/pulmonary embolism during their stay in psychiatric in-patient units, including dementia units and functional assessment units.

Inclusion criteria were as follows: individuals aged ≥65 years; individuals admitted to a psychiatric in-patient unit; individuals who developed DVT or pulmonary embolism, including those with a history of VTE; and individuals on prophylaxis for VTE during their hospital stay.

Exclusion criteria were as follows: individuals aged <65 years; those admitted to surgical wards or from within surgical or orthopaedic settings, as they represent an alternative patient group; and patients with trauma or recent fractures.

### Intervention

Studies that evaluated prophylaxis used to prevent VTE, either mechanical or low-molecular-weight heparin, were included. We reviewed studies for presence of VTE (DVT or pulmonary embolism) as main outcomes. We investigated study author, title, year, setting and number of participants. We also examined patient characteristics such as age, gender, comorbidities, prophylaxis and risk assessment.

### Data extraction

**Fig. 1:**
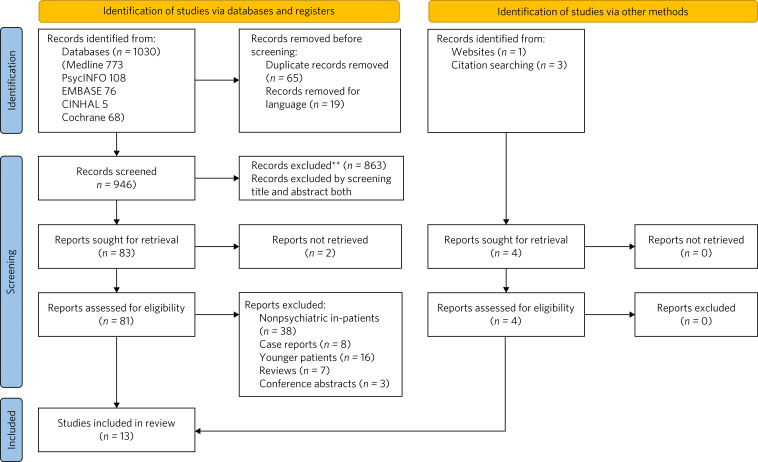
Preferred Reporting Items for Systematic reviews and Meta-Analyses (PRISMA) flowchart.

All articles were initially extracted into a Microsoft Excel spreadsheet. After screening through the titles and abstracts, 81 articles were retrieved for a detailed assessment. We excluded 72 articles for reasons as listed in the Preferred Reporting Items for Systematic reviews and Meta-Analyses (PRISMA) flowchart (Fig. 1). No discrepancies in numerical data were found. Quality assessments were conducted independently by two reviewers (D.C., T.C.), against the Quality Assessment Tool for Observational Cohort and Cross-sectional Studies,^19^ and disagreements resolved internally. The PRISMA flow diagram for papers in this review is presented in Fig. 1, and the checklist in Supplementary Fig. 1 available at https://doi.org/10.1192/bjb.2025.9.

## Results

### Search results

The search strategy identified 1030 articles, of which 19 were excluded as not being in English and 65 were duplicates. A total of 946 abstracts were further screened, and 83 articles retrieved. As noted in the PRISMA flowchart, 38 studies were further excluded as they had non-psychiatric patients and 16 studies included younger patients. There were seven reviews, three conference abstracts and eight case reports that did not meet our inclusion criteria. Two studies were not included as, despite all attempts, it was not possible to retrieve them from the searches. Only nine studies met all the criteria and were included in the review. More details of these studies are provided in the tables. Nine studies were initially identified.^5,20–27^ Four other studies were identified through cross-referencing articles.^6,12,28,29^ In total, we identified 13 studies that met our criteria.

Ten studies included patients from psychiatric in-patient units (summarised in Table 1), and three studies included patients from geriatric (physical health/acute care) units with psychiatric diagnoses (summarised in Table 2). Two studies were prospective in design,^21,26^ and the rest were retrospective in nature, with cross-sectional assessment of patient records. Two studies were part of local trust audits to assess factors associated with VTE in older in-patients.^5,20^ The ten studies from psychiatric units included a total sample size of 101 458 in comparison with 5780 identified from geriatric settings. Schmedt and Garbe^27^ had the highest sample size of 72 591, followed by Wang et al^12^ with 12 939, Gaertner et al^29^ with 6218 and Ishida et al^22^ with 5268. Other studies had lower sample sizes.

**Table 1:** Study characteristics of psychiatric in-patients

Study authors	Year	Country	Setting	Design	No. of patients	Age of patients in years	Gender	VTE occurrence rate	Diagnosis as a risk	Medication as a risk	Mobility as a risk	Risk assessment/ screening	Prophylaxis	Added comments
Liu et al^28^	2013	Australia	In-patients over 65 years	Retrospective audit	192	79.1 ± 10	60% Women	5/197 (2.6%)	Higher in depression and lower in dementia	No association with antipsychotics	Not mentioned	81.8% VTE screened	16.7% low-molecular-weight heparin	Dementia associated with reduced risk of VTE compared with those without dementia. Patients with depression had higher risk of VTE compared with those without depression
Ellis et al^20^	2019	UK	Psychiatric in-patient unit	Retrospective audit	470	Not mentioned (202 out of 470 above 60 years)	Not mentioned	Not calculated	Dementia	Not mentioned	Not mentioned	20 (4.3%) screened	14.9% had contraindication to prophylaxis	Association between dementia in patients on old-age wards and the risk of developing VTE
Delluc et al^21^	2012	France	Psychiatric in-patient unit	Prospective cohort study	471	Median 60 years	53.9% Female	2.5% on day 10 and 3.5% at day 90	High risk in dementia, not psychosis	Not associated with antipsychotic use but antidementia drugs were possible risk factor	Reduced mobility	All participants screened at admission	4 (0.8%) received prophylaxis	Incidence of VTE was higher in patients over 75 years, at 8.2 *v.* 3.5% as a combined all-age figure. Dementia as a risk factor for VTE only in patients over 75 years of age. Antidementia drugs was associated with risk of VTE
Ishida et al^22^	2021	Japan	Psychiatric in-patient unit	Cross-sectional case review	5268	Median age 47 years	51% males, higher in females	4.9% Prevalence	Depression and catatonia	Not recorded	Catatonia (subtype not recorded)	All patients underwent D-dimer and ultra sounds scan if positive	Not mentioned	Association between catatonia and VTE
Wang et al^12^	2023	China	Psychiatric in-patient units	Retrospective observational study	12 939	Cases median age 67 years	Female: male 1.4:1.0	1.21% (156)	Crude incidence rates of VTE; organic disorders 5.2%, mood disorders 1.07, psychosis 1.0%	No association	Not mentioned	Not done	Not mentioned	Patients at risk of VTE tend to be older if they are diagnosed as having psychosis. VTE occurrence was highest in patients with organic mental illness. Higher HRSD scores were associated with VTE risk. HRSA scores were found to be protective factors. Conventional risk assessment tools were not useful. Developed their own risk assessment tool
Gaertner et al^29^	2017	France	Psychiatric in-patient units	Retrospective case–control study	6218	Cases mean age 67.5 years	54% Males in cases group	47.8 cases per 1000 patient-years	No difference in two groups	No association	No significant association	Not mentioned	Not mentioned	
Yoshizawa et al^6^	2021	Japan	Psychiatric in-patient units	Retrospective cross-sectional case review	133	Median age 67 years	68% Female	10.5% Prevalence	Severity of depression	No association with antipsychotics or antidepressants	Not mentioned	All patients had D-dimer and ultrasound scan	1/133 for prophylaxis	10.5% of patients with depression developed VTE, all of whom were asymptomatic. Severity of depression rated by HRSD scores was associated with high risk of VTE
Takeshima et al^25^	2018	Japan	Psychiatric in-patient units	Retrospective case–control study	1681, of which 101 underwent CE CT scan	Mean 61.5 ± 16.0 years	60% Female	2.3% all patients, 61.1% in catatonia, 4.1% in restrained patients	Catatonia	No association with antipsychotic or antidepressants	Not associated with restrains	All suspected patients underwent D-dimer	56.4% received VTE prophylaxis	Catatonia was associated with increased risk of developing VTE. No association between antidepressant sue or dosage and VTE
Van Zyl et al^5^	2013	UK	Psychiatric old-age wards	Retrospective case review	1495	Mean 68 years	71% female	1.14%	Most common diagnoses: depression (29%) and vascular dementia (23%)	Not recorded	Not mentioned	No record of risk assessment	No record of prophylaxis	
Schmedt and Garbe^27^	2013	Germany	Review of statutory insurance record as a proxy for hospital admissions	A nested case–control cohort study	72 591	Mean 78.3 (s.d. 7.7) years	55.3% Female, women more at risk	5.6 per 1000 person-years	Patients with dementia on antipsychotics are at higher risk	VTE higher in current use of antipsychotic and concurrent users of first- and second-generation antipsychotics	Not reported	Included patients with diagnosed VTE	Not recorded	Increased risk of VTE for current antipsychotic users and concurrent users of second- and first-generation drugs. Odds ratio was significantly elevated for new users of antipsychotics who were commenced on medication within 90 days

VTE, venous thromboembolism; HRSD, Hamilton Rating Scale for Depression; HRSA, Hamilton Rating Scale for Anxiety; CE CT, contrast-enhanced computerised tomography.

**Table 2:** Study characteristics, geriatric settings

Study authors	Year	Country	Setting	Design	No. of patients	Age in years	Gender	VTE occurrence rate	Diagnosis as a risk	Medication as a risk	Mobility as a risk	Risk assessment/ screening	Prophylaxis	Added comments
Kleijer et al^23^	2010	The Netherlands	Record on community pharmacies on in-patient admissions	A case–control study nested within cohort of psychiatric patients	1031 Cases and 4125 control	76 (60–104)	43% female	0.90%	Not mentioned	No association with antipsychotics, or type or 5HT affinity	Not recorded	Included patients with VTE	Not recorded	Current use of antipsychotics was not associated with the risk of VTE in a nested cohort study of around 111 818 patients
Weill Engerer et al^26^	2004	France	Geriatric unit	A prospective case–control study	310 Cases and 310 controls	85.7 ± 7.0	75% Female	Not calculated	Not mentioned	Not mentioned	Increased duration and the type of immobility (settings)	Not reported (included symptomatic patients)	Not reported	VTE incidence higher in acute in-patient settings compared with long-term units. Odds of VTE increases 1.5-fold over 10 years. Restriction of mobility; both the type and duration of immobility were associated with patients developing VTE
Gallerani et al^24^	2012	Italy	Geriatric unit	Case series	4	Mean age 78.2 years	3 females	0.66% Pulmonary embolism	Not mentioned	Risperidone associated with risk of pulmonary embolism	Not mentioned	All patients had VTE	Not mentioned	

VTE, venous thromboembolism; 5HT, 5Hydroxy tryptophan.

We performed a quality assessment of the included studies, using the National Institutes of Health (NIH) Quality Assessment Tool for Observational Cohort and Cross-Sectional Studies.^19^ Because of the heterogeneity of the studies included and two studies being retrospective in nature, only 11 studies could be qualitatively assessed. The quality assessment is summarised in Table 3. Because of the cross-sectional nature of the studies, most studies were limited in their quality, with only three studies being good.^21,23,27^

**Table 3:** National Institutes of Health quality assessment tool for observational cohort and cross-sectional studies

Criteria	Gaertner et al^29^	Kleijer et al^23^	Delluc et al^21^	Weill-Engerer et al^26^	Ishida et al^22^	Wang et al^12^	Takeshima et al^25^	Yoshizawa et al^6^	Schmedt and Garbe^27^	Liu et al^28^	Ellis et al^20^
1. Was the research question or objective in this paper clearly stated?	Reported	Reported	Reported	Reported	Reported	Reported	Reported	Reported	Reported	Reported	Reported
2. Was the study population clearly specified and defined?	Reported	Reported	Reported	Reported	Reported	Reported	Reported	Reported	Reported	Reported	Reported
3. Was the participation rate of eligible persons at least 50%?	Reported	Reported	Reported	Reported	Reported	Reported	Reported	Reported	Reported	Reported	Reported
4. Were all the subjects selected or recruited from the same or similar populations (including the same time period)? Were inclusion and exclusion criteria for being in the study prespecified and applied uniformly to all participants?	Reported	Reported	Reported	Reported	Reported	Reported	Reported	Reported	Reported	Reported	Reported
5. Was a sample size justification, power description, or variance and effect estimates provided?	Not reported	Not reported	Not reported	Not reported	Not reported	Not reported	Not reported	Not reported	Not reported	Not reported	Not reported
6. For the analyses in this paper, were the exposure(s) of interest measured prior to the outcome(s) being measured?	Not reported	Not reported	Reported	Not reported	Not reported	Not reported	Not reported	Not reported	Reported	Not reported	Not reported
7. Was the timeframe sufficient so that one could reasonably expect to see an association between exposure and outcome if it existed?	Not reported	Reported	Reported	Not reported	Not reported	Not reported	Not reported	Not reported	Reported	Not reported	Not reported
8. For exposures that can vary in amount or level, did the study examine different levels of the exposure as related to the outcome (e.g., categories of exposure, or exposure measured as continuous variable)?	Not reported	Reported	Reported	Not applicable	Not reported	Not reported	Reported	Reported	Reported	Not reported	Not reported
9. Were the exposure measures (independent variables) clearly defined, valid, reliable, and implemented consistently across all study participants?	Reported	Reported	Reported	Not applicable	Reported	Reported	Reported	Reported	Reported	Reported	Not reported
10. Was the exposure(s) assessed more than once over time?	Not reported	Not reported	Reported	Not reported	Not reported	Not reported	Not reported	Not reported	Not reported	Not applicable	Not applicable
11. Were the outcome measures (dependent variables) clearly defined, valid, reliable, and implemented consistently across all study participants?	Reported	Reported	Reported	Not applicable	Reported	Reported	Reported	Reported	Reported	Cannot determine	Not reported
12. Were the outcome assessors blinded to the exposure status of participants?	Not applicable	Not reported	Not reported	Not applicable	Not applicable	Not applicable	Not applicable	Not applicable	Not applicable	Not applicable	Not applicable
13. Was loss to follow-up after baseline 20% or less?	Cannot determine	Not applicable	Reported	Not applicable	Not applicable	Not applicable	Not applicable	Not applicable	Cannot determine	Not applicable	Not applicable
14. Were key potential confounding variables measured and adjusted statistically for their impact on the relationship between exposure(s) and outcome(s)?	Not reported	Reported	Cannot determine	Reported	Not reported	Cannot determine	Not reported	Not reported	Reported	Not reported	Not reported

### Rates of VTE

Ten studies provide occurrence rates of VTE in their populations. In psychiatric units, VTE rates ranged from 0.9 to 10.5%.^12,21–23,25,28,29^ Delluc et al found the incidence of VTE was higher in patients older than 75 years, at 8.2%, compared with a rate of 3.5% across all age groups. Weil-Engerer et al found VTE incidence was higher in acute in-patient settings compared with long-term units.^21,26^ Yoshizawa et al found that 10.5% of patients with depression developed VTE, all of whom were asymptomatic.^6^ These high rates may be reflective of the authors screening all the patients for VTE, but may also possibly be a result of their stronger methodological approach. Table 4 shows comparison of sample sizes, VTE rates and risk factors between the studies from psychiatric and geriatric settings.

**Table 4:** Comparison between psychiatric and geriatric studies

	Psychiatric patients	Geriatric patients
Number of studies	Ten	Three
Total number of patients	101 458	5780
VTE rates	1.14–10.9%	0.66–0.9%
Risk factors	Dementia associated with VTE risk	Risk of VTE higher in acute units compared to long-term units
	Depression and catatonia identified as risk factors	Restriction of mobility associated with risk of VTE
	Antidepressants not associated with risk of VTE	Antipsychotics not reported as risk factor
	Antipsychotics identified as risk factor in only one out of ten studies	
	Anti-dementia drugs noted as putative risk factor for VTE	

VTE, venous thromboembolism.

### Age

Increasing age is an important risk factor for the development of VTE. Around 80% of studies included in this review note that older patients were at higher risk of developing VTE, and this was across both psychiatric and geriatric in-patient units.^12,20–23,26^ Weill-Engerer et al found the odds of VTE increased 1.5-fold over 10 years.^26^ Some risk factors were age-related, as one study reported dementia as a risk factor for VTE only in patients older than 75 years of age.^21^ Wang et al noted that patients at risk of VTE tended to be older if they were diagnosed as having psychosis.^12^ Interestingly, one study reported no association between age and the risk of developing VTE.^28^ This difference might have been because of its small sample size.

### Gender

Results were mixed regarding the association between gender and risk of VTE. Thus, four studies noted that women were at higher risk of developing VTE.^5,12,22,27^ Only one study reported higher rates of VTE in men (54%) compared with controls, but this result was not statistically significant.^29^ Four studies did not report a link between the gender of patients and their risk of VTE.^6,21,25,28^

### Diagnosis

Psychiatric illness by itself is associated with higher risk of developing VTE. Rates of VTE occurrence differ based on psychiatric diagnosis. Wang et al noted the occurrence was highest in patients with organic mental illness (5.6%), followed by mood disorder (1.07%), schizophrenia (1.1%) and other mental health diseases, including neurotic disorder and substance-induced mental health disorders.^12^ This was also reported by Delluc et al, who found that patients with dementia who were older than 75 years of age are were higher risk of developing VTE, with an incidence ranging from 8.2 to 12.5%.^21^ Additionally, there was an association between dementia in patients on old-age wards and the risk of developing VTE.^20^ In contrast, Liu et al found that wandering behaviour in dementia was associated with reduced risk of VTE, with 0.1% of patients with dementia developing VTE compared with 5.8% of patients without dementia (*P* < 0.07).^28^

Depression was found to be associated with VTE in three studies.^6,22,28^ Liu et al found patients with depression had higher risk of VTE compared with those without depression (6.3 *v.* 0.8%, *P* < 0.043).^28^ One study reported that the severity of depression as rated by Hamilton Rating Scale for Depression (HRSD) score was associated with high risk of VTE, with an odds ratio of 1.22 (95% CI 1.08–1.3; *P* < 0.001).^6^ In keeping with this, Wang et al reported that higher HRSD scores were associated with VTE risk, whereas the Hamilton Rating Scale for Anxiety (HRSA) scores were found to be protective factors.^12^

Catatonia was associated with increased risk of developing VTE. VTE was observed in 61.1% (11/18) of patients with catatonia, 4.1% (11/270) of restrained patients without catatonia and 1.2% (17/1393) of unrestrained patients without catatonia.^25^ This finding was replicated by Ishida et al, who also reported an association between catatonia and VTE, with an odds ratio of 5.84 (95% CI 2.69–12.70; *P* < 0.001).^22^ The remaining studies either did not record or did not identify any significant association between other mental illness and the risk of VTE.^23,29^

### Medication

The association between medication use and DVT reported mixed findings. In terms of antipsychotic use, most studies found no association between the use of antipsychotics and VTE.^12,20,22,23,25,28,29^ A nested case–control study of 111 818 patients with at least one antipsychotic drug prescription during 1998–2008 reported that current use of antipsychotics was not associated with the risk of VTE (odds ratio 0.9, 95% CI 0.7–1.1). The study found no difference between typical (chlorpromazine, chlorprothixene, periciazine, perphenazine, pipamperone) and atypical antipsychotics (risperidone, olanzapine, clozapine, quetiapine, aripiprazole, tiapride, sulpiride) in terms of risk of VTE.^23^ In contrast, a large study found significantly increased risk of VTE for current antipsychotic use (odds ratio 1.23, 95% CI 1.01–1.50) and concurrent use of second- and first-generation drugs (odds ratio 1.62, 95% CI 1.15–2.27). They also note that the odds ratio was significantly elevated for patients who were newly prescribed antipsychotics within 90 days (odds ratio 1.63, 95% CI 1.10–2.40) of commencement of medication, but not for those who were already using antipsychotics (odds ratio 1.09, 95% CI 0.87–1.36).^27^ Similarly, a case report of four patients who developed VTE while on risperidone noted an incidence rate of 5.2% in patients who received risperidone for a variety of psychiatric diagnoses.^24^

None of the studies included in this review found any association between the use of antidepressants and the risk of VTE.

Takeshima et al converted administered antidepressants in imipramine equivalents into three groups: 0 mg/day, >0 mg and <150/day, or >150 mg/day, and compared them with the risk of VTE, although they did not specify the antidepressants prescribed. Thirty-five patients had received at least one antidepressant, and they found no association between the dose of antidepressant and VTE.^25^ With regards to use of other psychotropic medications, one study reported that the use of antidementia drugs was associated with the risk of VTE, both at day 10 and day 90 of admission, but found no association between the use of anxiolytics and sedatives and VTE. In this study, individual drugs and their doses were not specified.^21^

### Mobility

Psychiatric patients can have immobility resulting from psychomotor retardation, catatonia, excess sedation or physical restraint. Three studies did not find any association between reduced physical mobility and the risk of VTE.^12,20,29^ Weil-Engerer et al found that restriction of mobility (both the type and duration of immobility) was associated with patients developing VTE.^26^ Additionally, Ishida et al noted that catatonia was associated with elevated risk of VTE in psychiatric in-patients.^22^ Takashima et al found that 61.1% (11/18) of patients with catatonia developed VTE, compared with only 4.1% (11/270) of restrained patients without catatonia and 1.2% (17/1393) of non-restrained patients without catatonia who developed VTE.^25^

### Risk assessment

Studies included in this review employed different methods in the assessment of risk factors for VTE. One local audit^20^ used the UK Department of Health VTE risk assessment tool. Liu et al^28^ used a risk assessment tool previously developed by Malý et al.^30^ Liu et al categorised patients into three groups (low, moderate and high, based on VTE scores); 81.8% (157/192) were assessed as low risk and 18.2% (35/192) as medium/high risk.^28^ Ishida et al developed their own screening tool with risk factors specific to psychiatric patients. This included factors such as age, gender, depressive episode, catatonia, active cancer, previous VTE and transfer from a general hospital, giving a score ranging from 0 to 14. A cut-off value of 2 had a sensitivity of 0.927, a specificity of 0.535 and a negative predictive value of 0.993. They reported that their tool was able to discriminate between patients with moderate to higher risk.^22^ Overall rates of screening and assessment for VTE were quite variable (5.9.24). Ellis et al reported that despite 30.6% of their sample being identified as at risk of VTE, only 4.3% had undergone risk assessment.^20^ This was even lower in an audit, where none of the patients underwent risk assessment at admission.^5^

### Prophylaxis

Most of the studies in this review either did not report use of thromboprophylaxis or the rates were low. In Delluc et al, only 0.4% of patients received prophylactic anticoagulation on the ward, whereas an audit found that none of their patients received any prophylactic measures.^5,21^ The highest rates were noted in a cross-sectional study, where 16.7% (32/192) received VTE prophylaxis. This included thromboembolic deterrent (thrombo–embolus deterrent hose stockings alone, unfractionated heparin/low-molecular-weight heparin, or thrombo–embolus deterrent stockings combined with unfractionated heparin/low-molecular-weight heparin).^28^

An audit in the UK recorded the reasons for not prescribing VTE prophylaxis for their patients. There were potential contraindications or concerns about prescribing VTE prophylaxis in 14.9% of patients. These included factors such as active self-harm, high risk of self- harm/previous self-harm and self-neglect, among others.^20^

## Discussion

We identified 13 studies that assessed the risk of VTE in older psychiatric in-patients. The occurrence and risk factors for VTE are quite heterogeneous. We found that the prevalence of VTE among psychiatric in-patient settings ranged from 1 to 11%, which is higher than those for community-dwelling older adults (range estimates between 0.14 and 0.8%).^31,32^ The rates we found were higher than those in a large cohort study that reported a hospital-acquired VTE incidence rate of 1.29%^33^ and a DVT prevalence of 2.4–9.6% in post-surgical patients.^34^ Our findings are consistent with previous reports that suggest higher VTE rates within hospital patients compared with community-dwelling patients.^35^

### Age as a risk factor

We found that age is the most important risk factor in the development of DVT, with older patients being at higher risk in all in-patient settings. Advancing age was found to be an important risk factor for VTE, and risk increased exponentially with age, as reported in few community-based studies.^32,34,35^ In a population-based study, the incidence tripled every 15 years.^32^ Weill-Engerer et al found that the odds of developing VTE increased by 1.5 times every 10 years.^26^ The differences could be attributed to factors such as prolonged immobility in older populations, more cardiovascular comorbidities, malignancy and greater length of hospital stay for older patients.^36–40^ Only one study, did not find any association between the age of the patient and risk of DVT, and this was from a single site and had a relatively small population sample size.^28^

### Diagnosis as a risk factor

Depression was found to be associated with higher risk of VTE in older patients, with severity of depression being proportionately linked with development of VTE. Similar results were found in other community-based studies.^8,41,42^ A metanalysis found that patients with depression were 1.31 times more likely to develop DVT. The higher risk for developing DVT in patients with depression may be attributable to factors like immobility, dehydration, poor nutrition and the use of antidepressants, although our review did not find risk associated with antidepressant drug use.^42^ Our review establishes the association of catatonia with the risk of developing VTE, a finding noted elsewhere too, albeit in smaller case reports.^43,44^ However, these reports were for younger patients, and further research is needed to ascertain the relationship between catatonia and VTE in older patients.

Three studies report that patients with dementia were at higher risk of developing VTE.^12,20,21^ Previous studies have reported that patients with dementia are at higher risk of VTE, and it is a leading cause of mortality in these patients; this may be attributable to prolonged immobility and pre-existing comorbidities.^45^ Only one study found that dementia was associated with reduced risk of VTE, with wandering behaviour acting as a protective factor.^28^

Although there are possible reported links between psychosis and VTE in the literature, these studies did not stratify patients according to their age groups.^16^ Our review in older patients shows only one study reporting such a link between the presence of psychotic symptoms and risk of developing VTE.^12^ This was the only study that specifically looked for association of psychiatric diagnosis (such as organic mental disorders, dementias, affective disorders and psychosis) with VTE.

### Medication use and VTE

The association between the use of antipsychotics and VTE risk is mixed. Two large-scale studies align with the findings of most of studies in this review, supporting that the use of antipsychotics is not associated with the risk of developing VTE.^46,47^ On the other hand, two studies found a relationship between antipsychotic use and a possible risk of developing pulmonary embolism, and note an increased risk of VTE in patients treated with antipsychotics.^24,27^ Some of the previous community-based studies noted that the risk of VTE is higher in older patients using atypical antipsychotics or combination of antipsychotics.^48,49^ A meta-analysis not specifically for older mental health in-patients concluded that irrespective of the type, use of antipsychotic increased the risk of developing VTE two-fold compared with the control population.^50^ It is possible that mixed findings regarding the risk of VTE may be because the studies had patients with psychosis, which may be a standalone risk factor for VTE, and thus causes overestimation of the association between antipsychotic use and VTE. In their review, Zhang et al also included studies with patients who were younger, with high heterogeneity, possibly leading to such differing results. It is also likely that younger patients with psychosis may be on higher dose of antipsychotics, leading to a dose–effect relationship between use of antipsychotics and the risk of VTE.^50^

None of the studies in this review found any association between the use and/or dose of antidepressants and the risk of VTE.^6,12,25,26^ This is in contrast to the finding that patients on antidepressants have a 1.22-fold risk of developing VTE, but the studies that reported such findings included a younger population or female patients only, and found it to be a class-specific effect.^42,51^ The studies included in this review did not compare the types and classes of antidepressant and the risk of VTE. One study reported that the use of antidementia drugs was associated with the risk of VTE.^21^ It is unclear whether this is an independent risk factor or a result of factors such as severity of dementia, presence of behavioural and psychological symptoms in dementia or the type of dementia itself.

### Immobility as a risk factor

Physical immobility is a recognised risk factor for the development of DVT in medical and surgical patients, and is usually not associated with mobile psychiatric patients. Reduced mobility was identified as risk factor in three of our studies,^22,25,26^ whereas three studies found no such association.^12,20,29^ A study reviewed listed various factors such as physical restraint, catatonia and psychomotor retardation as important risk factors for the development of VTE in psychiatric in-patient settings.^52^ One study found a close association between physical restraints and bedridden status with the risk of VTE and aspiration pneumonia. They found an association between retarded type of catatonia and risk of VTE, probably because of the combination of immobility and sedation.^53^ Two studies in this review identified catatonia as a potential risk factor for the development of VTE, especially if patients were also physically restrained.^22,25^

### Risk assessment and use of prophylaxis

In this review, we note that risk screening tools for VTE varied between settings. Two studies used existing VTE risk assessment tools, whereas one developed their own tools specifically for psychiatric patients.^20,22,28^ Screening tools like the Wells score, which have been quite useful in medical settings, were found to be less reliable in psychiatric settings. One study noted that 84% of VTE-positive patients were considered as low risk by their Wells score.^29^ Hence using a tool designed with psychiatry-specific risk factors in mind might be more useful. Ishida et al developed a tool that reportedly has a high sensitivity and negative predictive value, but its specificity is limited, and it needs wider research within mental health settings.^22^

The VTE screening rates in the studies varied from 0 to 17.9%, and is in keeping with quite variable screening rates ranging between 18 and 38% reported in older adult wards.^5,25,54,55^ This is in contrast to 95% screening rates for National Health Service (NHS) acute care provider wards and 98% for independent sector providers as reported by NHS England figures.^56^ Although guidelines recommend that all mental health patients have VTE risk assessments on admission, the rates of assessment for DVT on the wards remain low, and this was recognised by the National Institute for Health and Care Excellence (NICE) in its technology appraisal of 2018.^17^ Factors cited for low uptake include ward pressures, staff changes and practicalities of *pro forma* use or lack of awareness among staff.^54^

The rate of use of prophylactic anticoagulants reported in the studies conducted within mental health settings was very low (between 0 and 17.6%) in comparison with studies undertaken within acute care settings, where in more than one in two patients received DVT prophylaxis.^57^ In one of the studies, 14.9% of patients within mental health settings had potential contraindications or concerns about prescribing VTE prophylaxis despite 20.8% of these patients being considered as having increased VTE risk. Unlike in acute care settings, contraindications like risk of self-harm and self-neglect are unique to psychiatric settings.^20^ Another reason for such low rates is limited knowledge of pulmonary embolism compared with DVT.^54^ This differs from acute health care settings, where clinicians are more likely to be aware of cumulative risk factors, and hence more likely to administer prophylactic measures.^58^ This highlights the fact that that psychiatric patients warrant an individualised approach in screening and prophylaxis.

### Strengths and limitations of the study

To our knowledge, this is the only systematic review to explore the risk factors associated with VTE in older psychiatric in-patients. This review also included patients from different countries, and so may be more representative. Despite a robust review of literature, we could identify only nine studies that looked at patients in psychiatric settings. Most studies had mixed younger and older patients, and the results were not age-stratified. Three of the studies were from single centres, and their results may not be generalisable. Two of the studies were part of local Trust quality improvement studies, and they did not detail the important risk factors.

To conclude, there appears to be a complex relationship between various factors in the development of VTE in elderly psychiatric in-patients. The only risk factor that is consistent is advancing age. Diagnosis and psychotropic medication use were inconsistent risk factors. Studies that show an association of VTE with psychotropic medication use suggest that the greatest risk is within the first 90 days of drug treatment, and this increases with combination treatment. There is some evidence that other than the standard risk profiles already described for VTE, depression in older psychiatric in-patients and the presence of catatonia may be additional independent risk factors within older people's mental health in-patient settings. For patients with dementia, physical restraint may pose an added risk of VTE. Screening tools for VTE in older patients in mental health hospital settings are used at variable rates. Questionnaire-based tools used in medical and surgical settings have low positive predictive value in psychiatric settings, but using blood investigations like a D-dimer assay and imaging modalities in a mental health setting may be neither practical nor cost-efficient. Future research may involve the development of a screening tool tailored to screen the risk of VTE in psychiatric in-patients, as they represent a very specific group and are distinct from patients in medical and surgical units.

## Supporting information

Chari et al. supplementary material 1 Chari et al. supplementary material

Chari et al. supplementary material 2 Chari et al. supplementary material

## Data Availability

Data is available from the corresponding author, H.S., on reasonable request.

## About the authors

**Damodar Chari** is a specialist registrar in old age psychiatry at Leicestershire Partnership NHS Trust, Leicester, UK. **Tamara Chithiramohan** is a senior house officer in psychiatry at Leicestershire Partnership NHS Trust, Leicester, UK. **Ina Sawhney** is a consultant psychiatrist at Leicestershire Partnership NHS Trust, Leicester, UK. **Elizabeta B. Mukaetova-Ladinska** is a consultant psychiatrist at Leicestershire Partnership NHS Trust, Leicester, UK; and Professor of Old Age Psychiatry in the School of Psychology and Visual Sciences, University of Leicester, UK. **Lucy Beishon** is a higher specialist trainee in geriatric medicine at University Hospitals of Leicester NHS Trust, Leicester, UK; and an academic clinical lecturer in the Department of Cardiovascular Sciences, University of Leicester, UK. **Hari Subramaniam** is a consultant psychiatrist at Leicestershire Partnership NHS Trust, Leicester, UK; and an honorary senior lecturer in the School of Psychology and Visual Sciences, University of Leicester, UK.

## References

[1] Lozano R, Naghavi M, Foreman K, Lim S, Shibuya K, Aboyans V, et al. Global and regional mortality from 235 causes of death for 20 age groups in 1990 and 2010: a systematic analysis for the global burden of disease study 2010. Lancet 2012; 380(9859): 2095–128. 23245604 10.1016/S0140-6736(12)61728-0 PMC10790329

[2] Søgaard KK, Schmidt M, Pedersen L, Horváth-Puhó E, Sørensen HT 30-year mortality after venous thromboembolism: a population-based cohort study. Circulation 2014; 130(10): 829–36. 24970783 10.1161/CIRCULATIONAHA.114.009107

[3] Khan F, Tritschler T, Kahn SR, Rodger MA Venous thromboembolism. Lancet 2021; 398(10294): 64–77. 33984268 10.1016/S0140-6736(20)32658-1

[4] Chuang LH, Gumbs P, van Hout B, Agnelli G, Kroep S, Monreal M, et al. Health-related quality of life and mortality in patients with pulmonary embolism: a prospective cohort study in seven European countries. Qual Life Res Qual Life 2019; 28(8): 2111–24. 10.1007/s11136-019-02175-z PMC6620245 30949836

[5] Van Zyl M, Wieczorek G, Reilly J. Venous thromboembolism incidence in mental health services for older people: survey of in-patient units. The Psychiatrist 2013; 37(9): 283–5.

[6] Yoshizawa K, Takeshima M, Ishino S, Ogasawara M, Fujiwara D, Itoh Y, et al. Severity of depressive symptoms is associated with venous thromboembolism in hospitalized patients with a major depressive episode. Neuropsychiatr Dis Treat 2021; 17: 2955–63. 34584413 10.2147/NDT.S331409 PMC8464371

[7] Anand SS, Wells PS, Hunt D, Brill-Edwards P, Cook D, Ginsberg JS Does this patient have deep vein thrombosis? JAMA 1998; 279(14): 1094–9. 9546569 10.1001/jama.279.14.1094

[8] Goldhaber SZ. Risk factors for venous thromboembolism. J Am Coll Cardiol 2010; 56(1): 1–7. 20620709 10.1016/j.jacc.2010.01.057

[9] Shi Y, Wang T, Yuan Y, Su H, Chen L, Huang H. Silent pulmonary embolism in deep vein thrombosis: relationship and risk factors. Clin Appl Thromb Hemost 2022; 28: 10760296221131034. 36199255 10.1177/10760296221131034 PMC9537479

[10] Lutsey PL, Zakai NA. Epidemiology and prevention of venous thromboembolism. Nat Rev Cardiol Nat Rev Cardiol 2023; 20(4): 248–62. 36258120 10.1038/s41569-022-00787-6 PMC9579604

[11] Lacut K, Le Gal G, Couturaud F, Cornily G, Leroyer C, Mottier D, et al. Association between antipsychotic drugs, antidepressant drugs and venous thromboembolism: results from the EDITH case-control study. Fundam Clin Pharmacol 2007; 21(6): 643–50. 18034665 10.1111/j.1472-8206.2007.00515.x

[12] Wang Z, Yang Y, He X, Jiang X, Gao X, Liu P, et al. Incidence and clinical features of venous thromboembolism in inpatients with mental illness. Clin Appl Thromb Hemost 2023; 29: 10760296231160753. 36855268 10.1177/10760296231160753 PMC9986904

[13] Fu Y-H, Liu P, Xu X, Wang P-F, Shang K, Ke C, et al. Deep vein thrombosis in the lower extremities after femoral neck fracture: a retrospective observational study. J Orthop Surg (Hong Kong) 2020; 28(1): 2309499019901172. 10.1177/2309499019901172 31994963

[14] Ishida T, Katagiri T, Uchida H, Takeuchi H, Sakurai H, Watanabe K, et al. Incidence of deep vein thrombosis in restrained psychiatric patients. Psychosomatics 2014; 55(1): 69–75. 23845320 10.1016/j.psym.2013.04.001

[15] Therasse A, Persano HL, Ventura AD, Tecco JM. Incidence and prevention of deep vein thrombosis in restrained psychiatric patients. Psychiatr Danub 2018; 30(Suppl 7): 412–4. 30439815

[16] Wilkowska A, Kujawska-Danecka H, Hajduk A. Risk and prophylaxis of venous thromboembolism in hospitalized psychiatric patients. a review. Psychiatr Pol 2018; 52(3): 421–35. 30218559 10.12740/PP/78891

[17] National Institute for Health and Care Excellence (NICE). Venous Thromboembolism in Over 16s: Reducing the Risk of Hospital-Acquired Deep Vein Thrombosis or Pulmonary Embolism. NICE, 2018 (https://www.nice.org.uk/guidance/ng89). 29697228

[18] Schmidt M, Horvath-Puho E, Thomsen RW, Smeeth L, Sørensen HT. Acute infections and venous thromboembolism. J Intern Med 2012; 271(6): 608–18. 22026462 10.1111/j.1365-2796.2011.02473.x PMC3505369

[19] Downes MJ, Brennan ML, Williams HC, Dean RS. Development of a critical appraisal tool to assess the quality of cross-sectional studies (AXIS). BMJ Open 2016; 6: e011458. 10.1136/bmjopen-2016-011458 PMC5168618 27932337

[20] Ellis N, Grubb CM, Mustoe S, Watkins E, Codling D, Fitch S, et al. Venous thromboembolism risk in psychiatric in-patients: a multicentre cross-sectional study. BJPsych Bull 2019; 43: 255–9. 31030692 10.1192/bjb.2019.25 PMC12402912

[21] Delluc A, Montavon S, Canceil O, Carpentier M, Nowak E, Mercier B, et al. Incidence of venous thromboembolism in psychiatric units. Thromb Res 2012; 130(6): e283–8. 23092750 10.1016/j.thromres.2012.10.002

[22] Ishida T, Shibahashi K, Sugai S, Abe D, Hamabe Y, Kashiyama T, et al. Development of a risk stratification scoring system for deep vein thrombosis upon psychiatric admission. J Psychosom Res 2021; 147: 110540. 34102544 10.1016/j.jpsychores.2021.110540

[23] Kleijer BC, Heerdink ER, Egberts TC, Jansen PA, van Marum RJ. Antipsychotic drug use and the risk of venous thromboembolism in elderly patients. J Clin Psychopharmacol 2010; 30(5): 526–30. 20814323 10.1097/JCP.0b013e3181f0e87d

[24] Gallerani M, Imberti D, Mari E, Marra A, Manfredini R. Risperidone and pulmonary embolism: a harmful association? Case series and review of the literature. Acta Neuropsychiatr 2012; 24(6): 361–8. 25287179 10.1111/j.1601-5215.2012.00641.x

[25] Takeshima M, Ishikawa H, Shimizu K, Kanbayashi T, Shimizu T. Incidence of venous thromboembolism in psychiatric inpatients: a chart review. Neuropsychiatr Dis Treat 2018; 14: 1363–70. 29872303 10.2147/NDT.S162760 PMC5973315

[26] Weill-Engerer S, Meaume S, Lahlou A, Piette F, Saint-Jean O, Sachet O, et al. Risk factors for deep vein thrombosis in inpatients aged 65 and older: a case-control multicenter study. J Am Geriatr Soc 2004; 52(8): 1299–304. 15271117 10.1111/j.1532-5415.2004.52359.x

[27] Schmedt N, Garbe E. Antipsychotic drug use and the risk of venous thromboembolism in elderly patients with dementia. J Clin Psychopharmacol 2013; 33(6): 753–8. 24052055 10.1097/JCP.0b013e3182a412d5

[28] Liu X, O'Rourke F, Van Nguyen H. Venous thromboembolism in psychogeriatric in-patients–a study of risk assessment, incidence, and current prophylaxis prescribing. Int Psychogeriatr 2013; 25(6): 913–7. 23425864 10.1017/S1041610212002268

[29] Gaertner S, Piémont A, Faller A, Bertschy G, Hallouche N, Mirea C, et al. Incidence and risk factors of venous thromboembolism: peculiarities in psychiatric institutions. Int J Cardiol 2017; 248: 336–41. 28807508 10.1016/j.ijcard.2017.07.092

[30] Malý R, Masopust J, Hosák L, Konupcíková K. Assessment of risk of venous thromboembolism and its possible prevention in psychiatric patients. Psychiatry Clin Neurosci 2008; 62(1): 3–8. 18289135 10.1111/j.1440-1819.2007.01773.x

[31] Wendelboe AM, Raskob GE. Global burden of thrombosis: epidemiologic aspects. Circ Res 2016; 118(9): 1340–7. 27126645 10.1161/CIRCRESAHA.115.306841

[32] Naess IA, Christiansen SC, Romundstad P, Cannegieter SC, Rosendaal FR, Hammerstrøm J. Incidence and mortality of venous thrombosis: a population-based study. J Thromb Haemost 2007; 5(4): 692–9. 17367492 10.1111/j.1538-7836.2007.02450.x

[33] Neeman E, Liu V, Mishra P, Thai KK, Xu J, Clancy HA, et al. Trends and risk factors for venous thromboembolism among hospitalized medical patients. JAMA Netw Open 2022; 5(11): e2240373. 36409498 10.1001/jamanetworkopen.2022.40373 PMC9679881

[34] Danwang C, Temgoua MN, Agbor VN, Tankeu AT, Noubiap JJ. Epidemiology of venous thromboembolism in Africa: a systematic review. J Thromb Haemost 2017; 15(9): 1770–81. 28796427 10.1111/jth.13769

[35] Heit JA, Spencer FA, White RH. The epidemiology of venous thromboembolism. J Thromb Thrombolysis 2016; 41(1): 3–14. 26780736 10.1007/s11239-015-1311-6 PMC4715842

[36] Engbers MJ, van Hylckama Vlieg A, Rosendaal FR. Venous thrombosis in the elderly: incidence, risk factors and risk groups. J Thromb Haemost 2010; 8(10): 2105–12. 20629943 10.1111/j.1538-7836.2010.03986.x

[37] Ferrucci L, Cooper R, Shardell M, Simonsick EM, Schrack JA, Kuh D. Age-related change in mobility: perspectives from life course epidemiology and geroscience. J Gerontol A Biol Sci Med Sci 2016; 71(9): 1184–94. 26975983 10.1093/gerona/glw043 PMC4978365

[38] Folsom AR, Boland LL, Cushman M, Heckbert SR, Rosamond WD, Walston JD. Frailty and risk of venous thromboembolism in older adults. J Gerontol A Biol Sci Med Sci 2007; 62(1): 79–82. 17301042 10.1093/gerona/62.1.79

[39] Olsen H, Länne T. Reduced venous compliance in lower limbs of aging humans and its importance for capacitance function. Am J Physiol 1998; 275(3): H878–86. 9724292 10.1152/ajpheart.1998.275.3.H878

[40] Mackman N. New insights into the mechanisms of venous thrombosis. J Clin Invest 2012; 122(7): 2331–6. 22751108 10.1172/JCI60229 PMC3386811

[41] Lee CW, Liao CH, Lin CL, Liang JA, Sung FC, Kao CH. Depression and risk of venous thromboembolism: a population-based retrospective cohort study. Psychosom Med 2015; 77(5): 591–8. 25984821 10.1097/PSY.0000000000000193

[42] Kunutsor SK, Seidu S, Khunti K. Depression, antidepressant use, and risk of venous thromboembolism: systematic review and meta-analysis of published observational evidence. Ann Med 2018; 50(6): 529–37. 30001640 10.1080/07853890.2018.1500703

[43] Lachner C, Sandson NB. Medical complications of catatonia: a case of catatonia-induced deep venous thrombosis. Psychosomatics 2003; 44(6): 512–4. 14597687 10.1176/appi.psy.44.6.512

[44] Sapota-Zaręba K, Nasierowski T. Pulmonary embolism – a considerable clinical challenge in psychiatry. Case reports. Postep Psychiatr Neurol 2021; 30(4): 293–7. 37082557 10.5114/ppn.2021.111953 PMC9881643

[45] Nuñez MJ, Villalba JC, Cebrián E, Visoná A, Lopez-Jimenez L, Núňez M, et al. Venous thromboembolism in immobilized patients with dementia. Findings from the RIETE registry. Thromb Res 2012; 130(2): 173–7. 22374336 10.1016/j.thromres.2012.02.006

[46] Wolstein J, Grohmann R, Rüther E, Hippius H. Antipsychotic drugs and venous thromboembolism. Lancet 2000; 356(9225): 252. 10.1016/S0140-6736(05)74503-7 10963225

[47] Ray JG, Mamdani MM, Yeo EL. Antipsychotic and antidepressant drug use in the elderly and the risk of venous thromboembolism. Thromb Haemost 2002; 88(2): 205–9. 12195690

[48] Parker C, Coupland C, Hippisley-Cox J. Antipsychotic drugs and risk of venous thromboembolism: nested case-control study. BMJ 2010; 341: c4245. 20858909 10.1136/bmj.c4245

[49] Liperoti R, Pedone C, Lapane KL, Mor V, Bernabei R, Gambassi G. Venous thromboembolism among elderly patients treated with atypical and conventional antipsychotic agents. Arch Intern Med 2005; 165(22): 2677–82. 16344428 10.1001/archinte.165.22.2677

[50] Zhang R, Dong L, Shao F, Tan X, Ying K. Antipsychotics and venous thromboembolism risk: a meta-analysis. Pharmacopsychiatry 2011; 44(5): 183–8. 21739416 10.1055/s-0031-1280814

[51] Parkin L, Balkwill A, Sweetland S, Reeves GK, Green J, Beral V, et al. Antidepressants, depression, and venous thromboembolism risk: large prospective study of UK women. J Am Heart Assoc 2017; 6(5): e005316. 28515116 10.1161/JAHA.116.005316 PMC5524086

[52] Van Neste EG, Verbruggen W, Leysen M. Deep venous thrombosis and pulmonary embolism in psychiatric settings. Eur J Psychiatry 2009; 23: 1.

[53] Funayama M, Takata T. Psychiatric inpatients subjected to physical restraint have a higher risk of deep vein thrombosis and aspiration pneumonia. Gen Hosp Psychiatry 2020; 62: 1–5. 31734627 10.1016/j.genhosppsych.2019.11.003

[54] Croxford A, Clare A, McCurdy K. Introduction of a venous thromboembolism prophylaxis protocol for older adult psychiatric patients. BMJ Qual Improv Rep 2015; 4(1): u205852.w3226. 10.1136/bmjquality.u205852.w3226 PMC4645951 26734379

[55] Powell H, Jenkinson J. Venous thromboembolism (VTE) risk assessment completion in psychiatric inpatients. BJPsych Open 2021; 7(Suppl 1): S98–9.

[56] NHS England. *Venous Thromboembolism (VTE) Risk Assessment 2019/20*. NHS England, 2020 (https://www.england.nhs.uk/patient-safety/venous-thromboembolism-vte-risk-assessment-19-20/).

[57] Bosson JL, Labarere J, Sevestre MA, Belmin J, Beyssier L, Elias A, et al. Deep vein thrombosis in elderly patients hospitalized in subacute care facilities: a multicenter cross-sectional study of risk factors, prophylaxis, and prevalence. Arch Intern Med 2003; 163(21): 2613–8. 14638561 10.1001/archinte.163.21.2613

[58] Samama MM. An epidemiologic study of risk factors for deep vein thrombosis in medical outpatients: the Sirius study. Arch Intern Med 2000; 160(22): 3415–20. 11112234 10.1001/archinte.160.22.3415

