# Supplementary material for: Risk of venous thrombosis and pulmonary embolism in older in-patients with mental illness: systematic review
Source: BJPsych Bull. 2026 Feb;50(1):36–48. doi: 10.1192/bjb.2025.9 (PMC13150511; doi:10.1192/bjb.2025.9)
Supplement: Chari et al. supplementary material 1 — Chari et al. supplementary material [file S2056469425000099sup001.docx]

Appendix

Search terms

“Venous thrombosis” OR “deep vein thrombosis” OR “superficial vein thrombosis” OR “pulmonary embolism” OR “deep vein thrombosis-pulmonary embolism” AND “psychiatric inpatients” OR “elderly hospitalised patients” OR “older hospitalised patients” OR “geriatric inpatients” OR “psychiatric hospitalised patients” OR “psychogeriatric inpatients” OR “mentally ill inpatients” OR “mental health hospitalised patients” OR “mentally ill patients” OR “mentally ill elderly patients” OR “mentally ill hospitalised patients” OR “mentally ill geriatric patients” OR “mentally ill geriatric hospitalised patients” OR “psychogeriatric hospitalised patients” OR “mentally ill older patients/people”. “Anticoagulants in hospitalized patients” OR “DVT prophylaxis treatment in hospitalised patients” OR “mechanical prophylaxis in DVT”
